# The Use of Ambulatory Blood Pressure Monitoring As Standard of Care in Pediatrics

**DOI:** 10.3389/fped.2017.00153

**Published:** 2017-06-30

**Authors:** Caitlin G. Peterson, Yosuke Miyashita

**Affiliations:** ^1^Department of Pediatrics, University of Pittsburgh School of Medicine, Pittsburgh, PA, United States

**Keywords:** pediatric hypertension, ambulatory blood pressure monitoring, white coat hypertension, masked hypertension, hypertensive target organ damage

## Abstract

Hypertension (HTN) is a significant global health problem, responsible for 7.5 million deaths each year worldwide. The prevalence of HTN is increasing in the pediatric population likely attributed to the increase in childhood obesity. Recent work has also shown that blood pressure (BP) tends to track from childhood to adulthood including BP-related target organ damage. In the last 25–30 years, pediatric use of ambulatory blood pressure monitoring (ABPM) has been expanding mainly in the setting of initial elevated BP measurement evaluation, HTN therapy efficacy follow-up, and renal disease. However, there are many clinical areas where ABPM could potentially be used but is currently underutilized. This review summarizes the current knowledge and the uses of pediatric ABPM and explores clinical areas where it can be very useful both to detect HTN and its longitudinal follow-up. And thus, ABPM could serve as a critical tool to potentially prevent early cardiovascular mortality and morbidity in wide variety of populations. With solid data to support ABPM’s superiority over clinic BP measurements and these clinical areas for its expansion, ABPM should now be part of standard of care in BP evaluation and management in pediatrics.

## Introduction

The prevalence of hypertension (HTN) in children and adolescents ranges from 1 to 5% while prehypertension has been reported as high as 10% with rates of both HTN and prehypertension increasing over the past two decades (1, 2) Proper assessment of blood pressure (BP) in children and adolescents is important because pediatric BP is the strongest identified predictor for adult HTN (3, 4). Most recently, BP trajectory from childhood to young adulthood has been shown to be associated with target organ changes, specifically left ventricular mass index (LVMI) and carotid intima-media thickness (cIMT) (5). In the United States, approximately 32% of adults have HTN and worldwide, the prevalence of adult HTN is around 40% (6, 7). HTN contributes to 7.5 million deaths or 12.8% of all deaths globally per year (7). In addition to its morbidity and mortality, HTN costs $48 billion each year to the United States health-care system (8). Other common chronic diseases including coronary artery disease, renal disease, diabetes, and obesity are also adversely affected by and contribute to HTN, making it the primary risk factor-related cause of death worldwide (9). And thus, appropriate diagnosis and treatment of HTN in the pediatric population is important to prevent future cardiovascular disease and premature deaths as well as to decrease its economic burden.

Elevated BP in children and adolescents is often found during routine well child examinations or sports participation physicals. The National High Blood Pressure Education Program Working Group on High Blood Pressure in Children and Adolescents defines HTN in the Fourth Report as average systolic BP (SBP) and/or diastolic BP (DBP) ≥the 95th percentile for age, gender, and height on three or more occasions (10). Prehypertension is defined as average SBP and/or DBP between the 90th and 95th percentile. If the 90th percentile is higher than 120/80, which is the prehypertension threshold for adults, then 120/80 is also the threshold value used in adolescents (10). Clinic BP measurements have potential problems including improper technique, terminal digit preference, observer bias, and accommodation effect. In addition, BP measurements obtained in clinics may not necessarily reflect the true BP of an individual throughout the day while repeated ambulatory BP measurements using automatic devices may reflect more representative BP values and better risk stratification (11, 12). We now have pediatric ambulatory blood pressure monitoring (ABPM) practice guidelines and increasing clinical outcome data associated with ABPM parameters (13, 14). This review will briefly discuss the current use of pediatric ABPM and will have in-depth discussion on the state-of-the-art pediatric ABPM outcome data and promising clinical areas of expansion for ABPM to make a provocative argument for its use as standard of care in pediatric BP evaluation and management.

## ABPM Use in Pediatrics

In order to overcome the limitations of clinic BP measurements mentioned above, ABPM has been in use in children and adolescents in the last 25–30 years (15, 16). A detailed description of ABPM and equipment has been published by the American Heart Association (AHA) in 2008 (13), and interested readers are encouraged to review this publication. In brief, a monitor is worn in the child’s home environment, which should provide more accurate measurement of their true BP including during sleep. The device consists of a light-weight monitor and appropriately sized BP cuff worn on the non-dominant arm. Awake measurements occur every 15–20 min and sleep measurements occur every 20–30 min. Most authorities define a valid study as one with at least one valid reading per hour, and >40 BP readings in the 24 h period or >65% of all possible BP readings for a partial day study(13). Both oscillometric and auscultatory devices are available and validated for children (17). The largest cross-sectional pediatric ABPM study to formulate normative data used oscillometric devices (18, 19). Oscillometric devices usually have fewer erroneous readings and are easier to use than auscultatory devices (13). Mainly for these reasons, oscillometric ABPM devices are more commonly used in current pediatric clinical practice. During the study period, a diary is kept to record when the child is awake, asleep, and active in addition to any medication taken during the study period that may influence BP. ABPM may cause mild sleep disturbance in some children but is generally well tolerated (14).

When analyzing ABPM data, the mean SBP and DBP are calculated for the 24 h period as well as awake and sleep hours. BP load is calculated as the proportion of readings above threshold values expressed in percentages. In children and adolescents, threshold values are the 95th percentile of gender and height. ABPM is not performed in young children (<5 years old) because there are no normative data for this age group, and this age group is unlikely to tolerate the frequent BP measurements. Currently, indications for ABPM include confirming the diagnosis of HTN, evaluating for white coat hypertension (WCH) and masked hypertension (MH), assessing effectiveness of antihypertensive medication, and determining if symptoms can be attributed to drug-related hypotension (14). There are six categories of BP staging based on combination of clinic BP measurements and ABPM:

(1) BP is considered normal if clinic BP < 90th percentile, mean awake and sleep SBP and DBP < 95th percentile, and awake and sleep SBP and DBP loads are all <25%,
(2) WCH is when clinic BP ≥ 95th percentile and mean awake and sleep SBP and DBP < 95th percentile, and awake and sleep SBP and DBP loads are all <25%,
(3) Prehypertension is when clinic BP ≥ 90th percentile or >120/80 and mean awake and sleep SBP and DBP < 95th percentile, and awake or sleep SBP or DBP load is ≥25%,
(4) MH is when clinic BP < 95th percentile and mean awake or sleep SBP or DBP > 95th percentile, and awake or sleep SBP or DBP load is ≥25%,
(5) Ambulatory HTN is when clinic BP > 95th percentile and mean awake or sleep SBP or DBP > 95th percentile, and awake or sleep SBP or DBP load is 25–50%, and finally
(6) Severe ambulatory HTN is when clinic BP > 95th percentile and mean awake or sleep SBP or DBP > 95th percentile, and awake or sleep SBP or DBP load is >50%. These diagnosis categories are found in a table in the AHA 2014 Update (14).

## Pediatric ABPM Outcome Data

Adult ABPM studies have consistently reported superiority of ABPM parameters over clinic BP in predicting mortality, cardiovascular events, and target organ damage such as left ventricular hypertrophy (LVH) (20–22). In contrast to adults, there are no pediatric data available that correlate patient outcome with ABPM parameters largely due to very low incidence of mortality and cardiovascular events. However, there have been an increasing number of pediatric studies that have investigated ABPM parameters with target organ changes, including the heart, the arterial wall thickness, the kidneys, and central nervous system development. The following subsections will summarize published pediatric ABPM studies showing correlation between ABPM parameters with various target organ changes. This will be followed by subsections summarizing pediatric ABPM studies on WCH, MH, renal disease, and antihypertensive medication efficacy.

### Target Organ Damage

Table 1 summarizes the pediatric ABPM studies that have investigated its parameters with target organ changes and their references. Among the target organs, the most studied is the heart, specifically the correlation between ABPM parameters and LVMI, which is a representation of left ventricular mass normalized by body surface area. It is now well established that left ventricular mass starts to increase with increasing BP even in the pediatric age range (23, 24), and LVH is an established risk factor of cardiovascular events in adults (25). There are several studies that have demonstrated that ABPM parameters are superior to clinic BP measurements in predicting higher left ventricular mass. Sorof et al. demonstrated LVMI strongly correlating with ABPM parameters such as mean SBP and SBP load where there was no correlation with clinic BP measurements (26). Maggio et al. found systolic ambulatory HTN by ABPM in 48% of obese children where clinic BP was normal in 55% of these hypertensive children, and after adjustment, LVMI was only associated with 24 h SBP (27). Richey et al. demonstrated that ABPM parameters such as higher 24 h SBP load predicted greater likelihood of increasing LVMI (28). Increased cIMT has been established as an independent risk factor for strokes in adults (29). There are now multiple pediatric studies showing an association between abnormal ABPM parameters with increased cIMT (30, 31). Further, obesity has also been associated with increased cIMT, but Lande et al. demonstrated several ABPM parameters to correlate strongly with cIMT in an age, gender, and body mass index matched study (32). In adults, microalbuminuria has been shown to be a marker of HTN-induced renal injury (33). However, pediatric ABPM studies have not demonstrated ABPM parameters to independently predict presence of microalbuminuria in non-diabetic children and adolescents to date (34, 35).

**Table 1:** Summary of pediatric ambulatory blood pressure monitoring (ABPM) studies of association between its parameters and target organ damage.

Organ	Target organ damage marker	Correlation with ABPM parameters?	Reference
Heart	Left ventricular mass index	Yes	(23, 24, 26–28)
Arterial vessels	Carotid intima-media thickness	Yes	(30–32)
Kidneys	Microalbuminuria	No	(34, 35)
Central nervous system	Executive function tests	Yes	(37–40)

There is fairly extensive adult literature, which demonstrates that HTN is associated with poorer cognitive performance including learning and memory, executive functions; and visuospatial, visuoconstructional, psychomotor, and perceptual abilities, and chronic uncontrolled HTN predicts cognitive decline over time (36). More recently, there is emerging preliminary data, which suggest that children and adolescents with primary HTN are associated with neurocognitive deficits when compared to normotensive controls. Early studies in children on the link between HTN and cognitive function include a cross-sectional study that demonstrates children with elevated clinic SBP have an independent association with lower digit span test scores, which is a measure of short-term memory, attention, and concentration (37). This was followed by a matched longitudinal study of parental ratings of executive function of children with ABPM confirmed primary HTN. At the time of initial diagnosis, hypertensive children had lower parental rating of executive function compared to matched normotensive children (38). After 1 year of antihypertensive therapy, ABPM demonstrated improved BP in the hypertensive group, which also correlated with improvement in parental ratings of their executive function (39). Most recently, a multicentered study of neurocognition in children demonstrated that children with ABPM confirmed untreated primary HTN had lower performance on neurocognitive testing, in particular, on measures of memory, attention, and executive functions, compared to normotensive matched controls (40).

In summary, the above published studies suggest that proper diagnosis of HTN by ABPM in children and adolescents has the potential to prevent and/or to treat target organ damages, in particular, the heart, the arterial vessels, and the central nervous system, which may not be possible with clinic BP measurements alone.

### White Coat Hypertension

White coat hypertension in children and adolescents may be fairly common as studies have reported a wide range of prevalence of 13–52% of children with elevated clinic BP measurements (41–43). ABPM serves an important role in the initial evaluation of elevated BP measurement as misdiagnosis of HTN in patients with WCH may lead to unnecessary testing and treatment, which may cause adverse side effects and events and will add unnecessary health care cost. The clinical significance of WCH is not clear at this point in children and adolescents, but there are studies to suggest that it may represent a prehypertensive state. Pediatric ABPM studies suggest that WCH may result in intermediate target organ changes (41, 44). In a study where age, gender, and BMI were matched for ABPM confirmed WCH subjects with confirmed hypertensive subjects and normotensive subjects, the mean LVMI of WCH subjects was between that of normotensive and hypertensive subjects. The difference between the WCH subjects and normotensive subjects was statistically significant (45). Another study found that ABPM confirmed WCH subjects tended to have higher LVMI than normotensive subjects but lower than hypertensive subjects, although no statistically significant differences were found between the groups (41). There is currently no practice guideline on laboratory and imaging evaluation or subsequent follow-up of children and adolescents with WCH, but efforts are underway for longitudinal studies for children and adolescents with WCH.

### Masked HTN

The prevalence of MH in general pediatric population has been reported to be just under 10% (42, 46) and around 35–50% in children and adolescents who have risk factors for HTN such as chronic kidney disease (CKD) and post-coarctation of aorta repair (47–49). Currently, the mechanism of MH is still unclear. There are convincing data to suggest that left ventricular mass changes detected in children and adolescents with MH is very similar to those with sustained HTN (42, 46). Thus, identifying patients with MH is critical so target organ damages can be reversed and to potentially delay cardiovascular events. Diagnosis of MH is difficult since performing ABPM in all pediatric patients with normal clinic BP measurements is impractical. Identifying children with risk factors for MH with conditions discussed in sections below and/or family history of early HTN and cardiovascular events is critical since ABPM could potentially “unmask” their HTN and reduce cardiovascular risk factors. Similar to WCH, further studies are needed so that evidence-based practice guidelines could be formulated in order to identify which pediatric populations should undergo ABPM to identify MH.

### CKD and Renal Transplantation

One of the most important populations to properly diagnose HTN is children with CKD as HTN is a well-known modifiable risk factor for progression of CKD (47, 50–52). These patients can often be missed as having HTN because of an increased risk for MH compared to the general population (49). A cross-sectional analysis of CKD in Children cohort with CKD stage 2 through 4, ages 9 through 15 years, found 38% of participants had MH and 18% had HTN confirmed by ABPM (49). Seventeen percent of this cohort had LVH with 34% from the MH group and 20% with confirmed HTN with increase in LVH prevalence from 19 to 39% over a 2-year period (49). Most importantly, the reduction in the rate of progression of CKD by tight BP control was demonstrated by the Effect of Strict Blood Pressure Control and ACE Inhibition on the Progression of Chronic Renal Failure in Pediatric Patients (ESCAPE) trial where intensified ABPM-guided HTN therapy over a 5-year period led to a 35% reduction in subjects with 50% decline in renal function or progression to end stage renal disease (52).

Hypertension implication in the renal transplant children and adolescents is similar to CKD population including its high prevalence, cardiovascular significance, and prognosis of allograft. When ABPM was used in renal transplant patients, prevalence of MH was found to be around 25–45%, and prevalence of uncontrolled HTN among treated patients ranged between 18 and 82% with median prevalence of 53% suggestive of the importance of routine use of ABPM in post-renal transplant population (53). Yearly ABPM-guided HTN treatment in 22 renal transplant patients after median follow-up of 9 years showed 14 of 17 children with treated HTN had excellent BP control, and the prevalence of LVH was only 4.5% with no progression of cIMT (54). Last, tighter BP control by annual ABPM-guided therapy resulted in normotensive group to have stabilization of the allograft function at 2 years after transplantation, and in contrast, the hypertensive group had a statistically significant decline in allograft function suggestive of preservation of allograft function with better BP control (53).

Thus, based on these published reports in CKD and post-renal transplant children and adolescents, the use of ABPM appears to be critical in their longitudinal follow-up not only to properly detect HTN but also to treat their HTN to slow down the progression of CKD or to preserve their allograft function.

### Antihypertensive Medication Efficacy

Ambulatory blood pressure monitoring is not only an effective way to properly diagnose HTN at the onset but it is also an effective way to determine the efficacy of HTN therapy. Seeman et al. demonstrated that nearly half of the pediatric patients on antihypertensive medications were found to have uncontrolled HTN by ABPM parameters. In addition, 35% of this study population had reclassification of controlled or uncontrolled HTN after having ABPM from their original classification based on clinic BP measurements, and the prevalence of LVH was significantly higher in uncontrolled HTN group compared to controlled HTN group (55). Similarly, Halbach et al. found that performing ABPM resulted in 63% of treated hypertensive children to have their medication changed (56). Last, Flynn also reported 4 out of 7 patients requiring adjustment of antihypertensive medications after undergoing ABPM (57). Thus, these studies are convincing of the clinical usefulness of ABPM for longitudinal BP assessment in efficacy of treated hypertensive children and adolescents.

Ambulatory blood pressure monitoring’s superiority at detecting treatment-induced BP changes compared to clinic BP measurements makes it ideal for its use in pediatric antihypertensive medication clinical trials. Using the ESCAPE trial cohort, Gimpel et al. showed that SD of ABPM responses were up to 39% smaller than those of clinic BP measured responses. Using power analysis, they showed that depending on the magnitude of the aimed BP reduction, sample size could be reduced by 57–75% with the use of ABPM, which would be critical in reducing the number of hypertensive subjects receiving placebo drugs (58).

## Clinical Areas of Expansion for ABPM

The use of ABPM in children and adolescents has become more prevalent especially when evaluating to distinguish sustained HTN from WCH for elevated clinic BP measurements and in patients with kidney diseases including CKD and post-renal transplantation as discussed in the previous section. However, there is a wide spectrum of clinical areas where ABPM is not routinely used currently and where it could be very useful in detecting hypertensive patients. Table 2 demonstrates these conditions in which ABPM can potentially be utilized even if they have normal clinic BP measurements.

**Table 2:** Clinical areas of expansion for ambulatory blood pressure monitoring (ABPM).

Potential clinical areas where ABPM should be used to detect masked HTN
Coarctation of aorta
Non-renal solid organ transplantation
Hematopoietic transplantation
Diabetes mellitus
Obstructive sleep apnea
Turner syndrome
William syndrome
Neurofibromatosis, type I
Sickle cell disease

### Coarctation of Aorta

Coarctation of aorta is one of the more common causes of secondary HTN in pediatrics and makes up about 2% of all children and adolescents with HTN (59). More recent reports have also demonstrated long-term mortality and morbidity even after successful coarctation repair. The survival rates of coarctation repair patients have been reported to be up to 20% less compared to age- and gender-matched populations mainly due to cardiovascular disease (60), and rates of persistent aortic hypoplasia and HTN have been reported to be as high as 20–48 and 21–42%, respectively (61, 62). Further, even in post-coarctation repair patients where the aortic arch were reported to be normal, 27% had hypertensive clinic BP measurements, 61% had HTN diagnosed by ABPM, and 55% had LVH with higher rates of LVH in ABPM confirmed hypertensive group compared to normotensive group (63). Therefore, ABPM appears to be a critical tool in following BP in post-coarctation of aorta repair patients (60, 63).

### Non-Renal Solid Organ Transplantation

The literature on the use of ABPM is scarce in non-renal solid organ transplantation. Because of the frequent uses of glucocorticoids and nephrotoxic agents such as calcineurin inhibitors and higher prevalence of CKD, HTN has been reported to be significantly higher than the general pediatric population in liver, intestinal, and heart transplantation (64–67). In the adult cardiac transplantation data registry, HTN incidence is 90% at 5 years after transplantation, and it is one of the main risk factors for cardiac allograft vasculopathy, the fourth leading cause of death at 10 years after transplantation (68). In adult liver transplant patients, cardiovascular disease is the second most common cause of non-liver death behind cancer (69). There have been a small number of pediatric ABPM studies in liver and heart transplant recipients, and they have concluded that clinic BP readings poorly correlate with ABPM parameters (70, 71). Therefore, ABPM should be strongly considered as part of routine posttransplant surveillance evaluation for precise diagnosis of HTN so that onset of cardiovascular mortality and morbidity may be delayed. This is also a fertile area for future and larger studies to investigate the exact prevalence of HTN in these high risk groups.

### Hematopoietic Cell Transplantation

Similarly, survivors of hematopoietic cell transplantation patients have been reported to have higher risk of HTN from multifactorial etiologies including the use of glucocorticoids, nephrotoxic medications including calcineurin inhibitors and various chemotherapy agents, total body irradiation, graft versus host disease, and kidney injury (72, 73). In a late mortality analysis of hematopoietic cell transplantation patients with median age of 25.9 years old, these survivors have a 2.3-fold increase in risk for premature cardiovascular related death (74). Seventy percent of children and adults have HTN during the first 2 years after hematopoietic cell transplant (72). Again, ABPM could be very useful aspect of routine posttransplant surveillance evaluation for the same reasons as the solid organ transplant recipients.

### Type I Diabetes Mellitus

Ambulatory blood pressure monitoring also appears to be promising tool in following pediatric patients with type I diabetes mellitus (T1DM). Children with T1DM were frequently found to have ABPM confirmed HTN with most of them being nocturnal HTN suggestive of the important role of ABPM in this population (75). Perhaps more clinically relevant, there was an independent association between nocturnal HTN detected on ABPM with development of microalbuminuria, which is a marker of diabetic nephropathy, in a cohort of adolescents and young adults with T1DM followed for more than 5 years (76). Combining the findings of these studies, ABPM could be used routinely in children and adolescents with T1DM not only to diagnose HTN earlier but also to use it longitudinally for more strict nocturnal BP control to prevent or to slow the progression of diabetic nephropathy.

### Obstructive Sleep Apnea

One of the more common comorbidity of obesity and obesity-related HTN is obstructive sleep apnea, and the use of ABPM appears to be helpful in this population. In a tertiary care hospital-based population, there was an independent association between apnea–hypopnea index and nighttime SBP and mean arterial pressure adjusting for adiposity variables suggestive of children with moderate-to-severe obstructive sleep apnea having higher ABPM parameters compared with children who were primary snorers (77). Similarly, in children with apnea–hypopnea index >5/h, ABPM parameters such as 24 h mean BP and BP loads were significantly increased, and perhaps more clinically relevant, early morning BP surge was more likely to occur in these children (78). Thus, ABPM could be very useful in detecting these abnormal BP patterns even if they have normal clinic BP in otherwise high-risk group of children and adolescents for future cardiovascular disease. Although not completely curative, adenotonsillectomy improved some of the ABPM parameters (79), suggestive of improvement in obstructive sleep apnea having contribution to lowering of BP. Best to our knowledge, although no pediatric study to date has shown lowering of BP with continuous positive airway pressure, adult literature has shown significant decreases in daytime and nighttime SBP and DBP with improvement in number of patients with prehypertension from 94 to 55% and MH from 30 to 5% (80).

### Other Pediatric Conditions with HTN Association

There are relatively common conditions in children and adolescents known to increase the risk of HTN including polycystic ovary syndrome, William syndrome, Neurofibromatosis type I, and Turner Syndrome. Single centered studies in each of these conditions have identified higher detection rates of HTN by ABPM compared to clinic BP measurements (81–85). Women with polycystic ovary syndrome have been found to have increased cardiovascular disease risk factors earlier in life (86), while women with Turner syndrome have a markedly increased incidence of ischemic heart disease, HTN, and stroke (87). Both children and adults with Williams syndrome have been found to have increased cardiovascular disease risk factors (82, 88). Traditionally, patients with sickle cell disease were observed to have lower clinic BP measurements largely attributed to poor urinary concentrating ability. However and surprisingly, a cross-sectional analysis of children with sickle cell disease found 10% to have elevated clinic BP measurements and 44% to have ABPM diagnosed HTN suggestive of high rates of MH in this population. Further, 59% of subjects were found to have impaired SBP nocturnal dipping and 13% had reversed nocturnal dipping (89).

In summary, there are a relatively large number of clinical areas discussed in this section where ABPM is currently underutilized but should be used routinely to detect MH and to follow BP longitudinally in order to prevent and to delay the onset of cardiovascular disease in growing number of high risk children and adolescents with chronic conditions. The 2016 European Society of HTN guidelines for the management of high BP in children and adolescents recommends ABPM for pediatric patients with diabetes mellitus type 1 and 2, CKD, solid organ transplants including renal, liver and heart, and severe obesity with or without sleep disordered breathing (90).

## Cost Effectiveness of ABPM

Traditional practice in pediatrics for a new diagnosis of HTN often included extensive evaluation of secondary causes of HTN including laboratory investigation, imaging studies, and target organ damage assessment, most often echocardiogram. A cost analysis at a pediatric HTN clinic where the protocol was to perform ABPM on all patients with stage I HTN clinic BP measurements found projected saving of $2.4 million for every 1,000 patients given their WCH rate of 46% (91). The cost savings were mainly attributed to not doing renal ultrasonography and echocardiogram on children with WCH, and authors speculated that the ABPM cost savings could be even higher as laboratory testing and number of clinic visits would be reduced for children with WCH. Davis et al. found that universal ABPM use at a pediatric HTN referral center accrued the lowest average charge per hypertensive youth identified and concluded that its universal use may be the most economically and clinically efficient diagnostic strategy (92).

## Limitations of ABPM

There are some limitations in our current knowledge and clinical use of pediatric ABPM. The most glaring is the need for more comprehensive normative ABPM data (14, 90). The ABPM normalized data being used today are based on values from the German Working Group on Pediatric HTN, which evaluated 1,141 children aged 5–20 years (18, 19). These children were a homogeneous Caucasian central European population, which is likely not generalizable in children of different races worldwide, and thus, normative data based on larger and multiethnic cohort are greatly needed. Another area in need of improvement is the development of BP measurement device that can measure DBP more accurately as this German normative data have almost flat DBP curve across all height in both genders (93).

Ambulatory blood pressure monitoring monitor availability in pediatric clinics is currently suboptimal. Based on a recent on-line survey among pediatric nephrologists at centers belonging to the Midwest Pediatric Nephrology Consortium in North America, 94% of the survey responders were at centers where ABPM was available, but 57% of responders stated that patients sometimes had to wait to have ABPM as monitors were not available at the time of the visits. This inconvenience is likely attributable to approximately 75% of survey responders practicing at centers owning <10 monitors (publication in press). Last, another potential barrier is the cost to start an ABPM program and its income potential. One ABPM monitor along with the software costs around $3,500–$4,000. In the United States, many government-based and other private health insurances either do not cover or have fairly low reimbursement rate for ABPM for children despite the cost effectiveness mentioned above and ABPM published guidelines starting in 2008. It is estimated that 190–200 ABPM studies would need to be performed to recover the initial starting cost (94). With wide spectrum of potential clinical areas of expansion as discussed above, better monitor availability at pediatric HTN clinics will be vital in ABPM becoming routine part of BP evaluation and management.

## Conclusion

**Figure 1:**
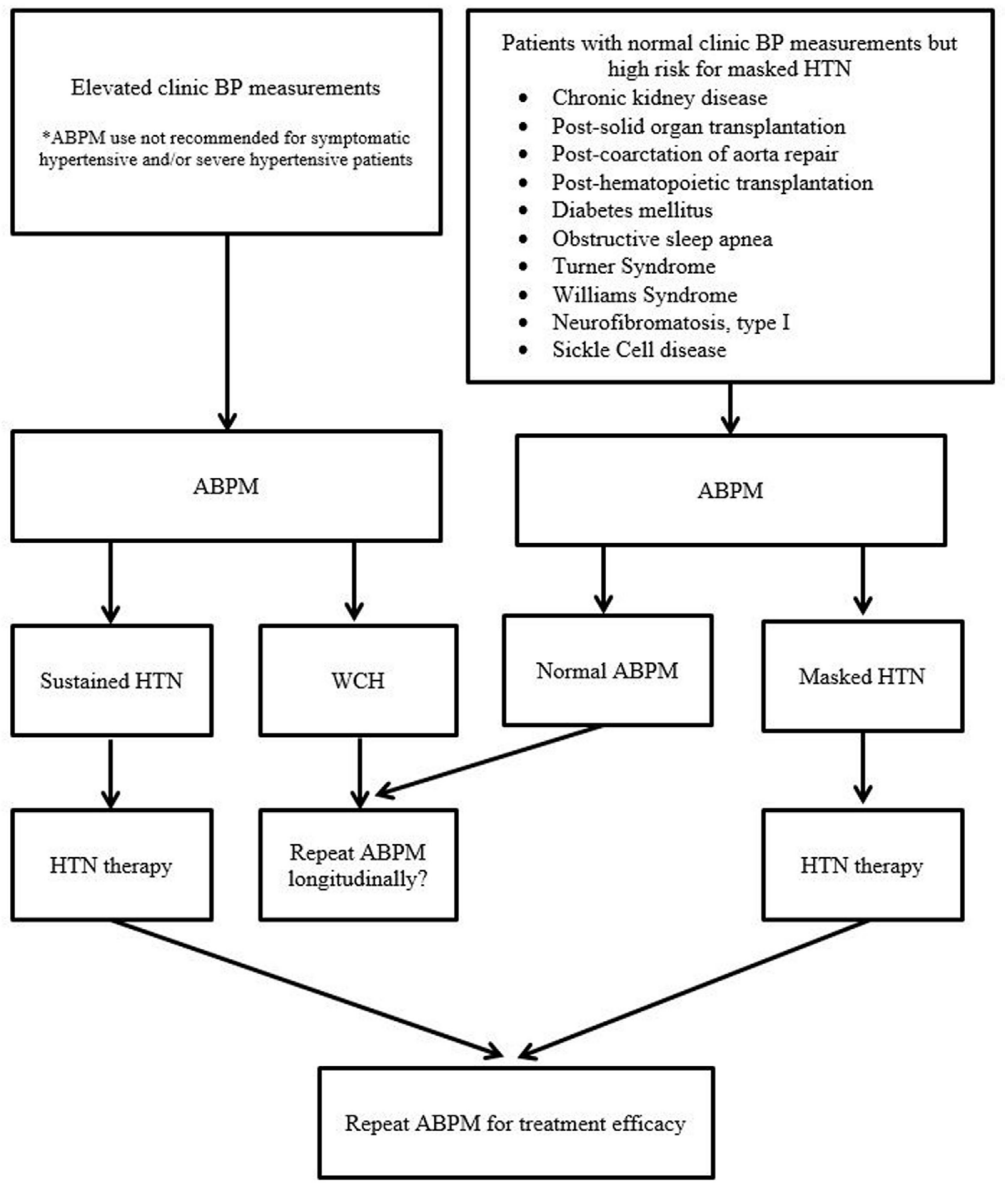
Schematic diagram of various points of ABPM utility in pediatric hypertension evaluation and management (BP, blood pressure; HTN, hypertension; ABPM, ambulatory blood pressure monitoring; WCH, white coat hypertension).

The health and economic burden of HTN is unquestioned worldwide. Based on recent data to suggest BP tracking from childhood to adulthood with target organ damages, earlier and proper diagnosis of HTN by ABPM is vital in the first step to prevent and to delay cardiovascular consequences of HTN. By properly identifying children and adolescents with WCH, ABPM can also reduce the number of children and adolescents undergoing unnecessary medical tests and experiencing adverse effects from unnecessary therapy. Routine use of ABPM appears to also save costs to the health-care system. Pediatric ABPM studies in the last 25–30 years are starting to demonstrate its superiority in target organ outcomes over clinic BP measurements. In addition, there are multiple clinical areas where ABPM use is currently infrequent, but its use may be very valuable in longitudinal care of many children and adolescents with chronic conditions. Figure 1 is a schematic diagram of various points where ABPM can be utilized in pediatric HTN. Certainly, we currently have both scientific and practical shortcomings in the pediatric use of ABPM, but this also provides wealth of future research target areas. Thus, in moving forward, we advocate ABPM to be standard of care in the field of pediatric HTN in both clinical and research settings.

## Author Contributions

CP and YM performed comprehensive literature search, drafting and editing of manuscript, final approval of the version, and agreed to be accountable for all aspects of the work.

## Conflict of Interest Statement

The authors declare that the research was conducted in the absence of any commercial or financial relationships that could be construed as a potential conflict of interest.

## References

[1] McNiece KL Poffenbarger TS Turner JL Franco KD Sorof JM Portman RJ. Prevalence of hypertension and pre-hypertension among adolescents. J Pediatr (2007) 150(6):640–4, 644.e1. 10.1016/j.jpeds.2007.01.052 17517252

[2] Sorof JM Lai D Turner J Poffenbarger T Portman RJ. Overweight, ethnicity, and the prevalence of hypertension in school-aged children. Pediatrics (2004) 113(3 Pt 1):475–82. 10.1542/peds.113.3.475 14993537

[3] Lauer RM Clarke WR. Childhood risk factors for high adult blood pressure: the Muscatine study. Pediatrics (1989) 84(4):633–41. 2780125

[4] Theodore RF Broadbent J Nagin D Ambler A Hogan S Ramrakha S Childhood to early-midlife systolic blood pressure trajectories: early-life predictors, effect modifiers, and adult cardiovascular outcomes. Hypertension (2015) 66(6):1108–15. 10.1161/HYPERTENSIONAHA.115.05831 26558818 PMC4646716

[5] Hao G Wang X Treiber FA Harshfield G Kapuku G Su S. Blood pressure trajectories from childhood to young adulthood associated with cardiovascular risk: results from the 23-year longitudinal Georgia stress and heart study. Hypertension (2017) 69(3):435–42. 10.1161/HYPERTENSIONAHA.116.08312 28093467 PMC5300982

[6] Nwankwo T Yoon SS Burt V Gu Q Hypertension among adults in the United States: National Health and Nutrition Examination Survey, 2011–2012. NCHS Data Brief (2013) 133:1–8. 24171916

[7] Raised Blood Pressure Situation and Trends. Global Health Observatory (GHO) Data. (2017). Available from: http://www.who.int/gho/ncd/risk_factors/blood_pressure_prevalence_text/en/

[8] Centers for Disease Control and Prevention; National Center for Health Statistics. Underlying Cause of Death 1999–2013 on CDC WONDER Online Database. (2015). Available from: http://wonder.cdc.gov/ucd-icd10.html

[9] World Health Organization. Global Health Risks: Mortality and Burden of Disease Attributable to Selected Major Risks. (2009). Available from: http://www.who.int/iris/handle/10665/44203

[10] National High Blood Pressure Education Program Working Group on High Blood Pressure in Children and Adolescents. The fourth report on the diagnosis, evaluation, and treatment of high blood pressure in children and adolescents. Pediatrics (2004) 114(2 Suppl):555–76. 10.1542/peds.114.2.S2.555 15286277

[11] Di Rienzo M Parati G Pomidossi G Veniani M Pedotti A Mancia G. Blood pressure monitoring over short day and night times cannot predict 24-hour average blood pressure. J Hypertens (1985) 3(4):343–9. 10.1097/00004872-198508000-00006 4045187

[12] Littler WA Honour AJ Pugsley DJ Sleight P Continuous recording of direct arterial pressure in unrestricted patients. Its role in the diagnosis and management of high blood pressure. Circulation (1975) 51(6):1101–6. 10.1161/01.CIR.51.6.1101 1132100

[13] Urbina E Alpert B Flynn J Hayman L Harshfield GA Jacobson M Ambulatory blood pressure monitoring in children and adolescents: recommendations for standard assessment: a scientific statement from the American Heart Association Atherosclerosis, Hypertension, and Obesity in Youth Committee of the council on cardiovascular disease in the young and the council for high blood pressure research. Hypertension (2008) 52(3):433–51. 10.1161/HYPERTENSIONAHA.108.190329 18678786

[14] Flynn JT Daniels SR Hayman LL Maahs DM McCrindle BW Mitsnefes M Update: ambulatory blood pressure monitoring in children and adolescents: a scientific statement from the American Heart Association. Hypertension (2014) 63(5):1116–35. 10.1161/HYP.0000000000000007 24591341 PMC4146525

[15] Garrett BN Salcedo JR Thompson AM. The role of ambulatory blood pressure monitoring in the evaluation of adolescent hypertension. Clin Exp Hypertens A (1985) 7(2–3):227–34. 4006237 10.3109/10641968509073542

[16] Portman RJ Yetman RJ West MS. Efficacy of 24-hour ambulatory blood pressure monitoring in children. J Pediatr (1991) 118(6):842–9. 10.1016/S0022-3476(05)82193-6 2040918

[17] Sphygmomanometers for Ambulatory Blood Pressure Measurement. (2014). Available from: http://www.dableducational.org/sphygmomanometers/devices_3_abpm.html

[18] Soergel M Kirschstein M Busch C Danne T Gellermann J Holl R Oscillometric twenty-four-hour ambulatory blood pressure values in healthy children and adolescents: a multicenter trial including 1141 subjects. J Pediatr (1997) 130(2):178–84. 10.1016/S0022-3476(97)70340-8 9042117

[19] Wuhl E Witte K Soergel M Mehls O Schaefer F German Working Group on Pediatric Hypertension. Distribution of 24-h ambulatory blood pressure in children: normalized reference values and role of body dimensions. J Hypertens (2002) 20(10):1995–2007. 10.1097/00004872-200210000-00019 12359978

[20] Clement DL De Buyzere ML De Bacquer DA de Leeuw PW Duprez DA Fagard RH Prognostic value of ambulatory blood-pressure recordings in patients with treated hypertension. N Engl J Med (2003) 348(24):2407–15. 10.1056/NEJMoa022273 12802026

[21] Verdecchia P Porcellati C Schillaci G Borgioni C Ciucci A Battistelli M Ambulatory blood pressure. An independent predictor of prognosis in essential hypertension. Hypertension (1994) 24(6):793–801. 10.1161/01.HYP.24.6.793 7995639

[22] Dolan E Stanton A Thijs L Hinedi K Atkins N McClory S Superiority of ambulatory over clinic blood pressure measurement in predicting mortality: the Dublin outcome study. Hypertension (2005) 46(1):156–61. 10.1161/01.HYP.0000170138.56903.7a 15939805

[23] Daniels SR Loggie JM Khoury P Kimball TR. Left ventricular geometry and severe left ventricular hypertrophy in children and adolescents with essential hypertension. Circulation (1998) 97(19):1907–11. 10.1161/01.CIR.97.19.1907 9609083

[24] Hanevold C Waller J Daniels S Portman R Sorof J International Pediatric Hypertension Association. The effects of obesity, gender, and ethnic group on left ventricular hypertrophy and geometry in hypertensive children: a collaborative study of the International Pediatric Hypertension Association. Pediatrics (2004) 113(2):328–33. 10.1542/peds.113.2.328 14754945

[25] Levy D Garrison RJ Savage DD Kannel WB Castelli WP. Prognostic implications of echocardiographically determined left ventricular mass in the Framingham Heart study. N Engl J Med (1990) 322(22):1561–6. 10.1056/NEJM199005313222203 2139921

[26] Sorof JM Cardwell G Franco K Portman RJ. Ambulatory blood pressure and left ventricular mass index in hypertensive children. Hypertension (2002) 39(4):903–8. 10.1161/01.HYP.0000013266.40320.3B 11967247

[27] Maggio AB Aggoun Y Marchand LM Martin XE Herrmann F Beghetti M Associations among obesity, blood pressure, and left ventricular mass. J Pediatr (2008) 152(4):489–93. 10.1016/j.jpeds.2007.10.042 18346502

[28] Richey PA Disessa TG Hastings MC Somes GW Alpert BS Jones DP. Ambulatory blood pressure and increased left ventricular mass in children at risk for hypertension. J Pediatr (2008) 152(3):343–8. 10.1016/j.jpeds.2007.07.014 18280838 PMC2763428

[29] O’Leary DH Polak JF Kronmal RA Manolio TA Burke GL Wolfson SK Jr. Carotid-artery intima and media thickness as a risk factor for myocardial infarction and stroke in older adults. Cardiovascular Health study Collaborative Research Group. N Engl J Med (1999) 340(1):14–22. 10.1056/NEJM199901073400103 9878640

[30] Litwin M Niemirska A Sladowska J Antoniewicz J Daszkowska J Wierzbicka A Left ventricular hypertrophy and arterial wall thickening in children with essential hypertension. Pediatr Nephrol (2006) 21(6):811–9. 10.1007/s00467-006-0068-8 16565870

[31] Stabouli S Kotsis V Papamichael C Constantopoulos A Zakopoulos N. Adolescent obesity is associated with high ambulatory blood pressure and increased carotid intimal-medial thickness. J Pediatr (2005) 147(5):651–6. 10.1016/j.jpeds.2005.06.008 16291358

[32] Lande MB Carson NL Roy J Meagher CC. Effects of childhood primary hypertension on carotid intima media thickness: a matched controlled study. Hypertension (2006) 48(1):40–4. 10.1161/01.HYP.0000227029.10536.e8 16735644

[33] Bigazzi R Bianchi S Baldari D Campese VM. Microalbuminuria predicts cardiovascular events and renal insufficiency in patients with essential hypertension. J Hypertens (1998) 16(9):1325–33. 10.1097/00004872-199816090-00014 9746120

[34] Conkar S Yilmaz E Hacikara S Bozabali S Mir S. Is daytime systolic load an important risk factor for target organ damage in pediatric hypertension? J Clin Hypertens (Greenwich) (2015) 17(10):760–6. 10.1111/jch.12608 26140344 PMC8031531

[35] Karpettas N Nasothimiou E Kollias A Vazeou A Stergiou GS. Ambulatory and home blood pressure monitoring in children and adolescents: diagnosis of hypertension and assessment of target-organ damage. Hypertens Res (2013) 36(4):285–92. 10.1038/hr.2012.220 23344131

[36] Waldstein SR Elias MF Neuropsychology of Cardiovascular Disease. Mahwah, NJ: Lawrence Erlbaum Associates (2001).

[37] Lande MB Kaczorowski JM Auinger P Schwartz GJ Weitzman M Elevated blood pressure and decreased cognitive function among school-age children and adolescents in the United States. J Pediatr (2003) 143(6):720–4. 10.1067/S0022-3476(03)00412-8 14657815

[38] Lande MB Adams H Falkner B Waldstein SR Schwartz GJ Szilagyi PG Parental assessments of internalizing and externalizing behavior and executive function in children with primary hypertension. J Pediatr (2009) 154(2):207–12. 10.1016/j.jpeds.2008.08.017 18823913 PMC2633107

[39] Lande MB Adams H Falkner B Waldstein SR Schwartz GJ Szilagyi PG Parental assessment of executive function and internalizing and externalizing behavior in primary hypertension after anti-hypertensive therapy. J Pediatr (2010) 157(1):114–9. 10.1016/j.jpeds.2009.12.053 20227722 PMC2904985

[40] Lande MB Batisky DL Kupferman JC Samuels J Hooper SR Falkner B Neurocognitive function in children with primary hypertension. J Pediatr (2017) 180:148–55.e1. 10.1016/j.jpeds.2016.08.076 27692987 PMC5183510

[41] Kavey RE Kveselis DA Atallah N Smith FC. White coat hypertension in childhood: evidence for end-organ effect. J Pediatr (2007) 150(5):491–7. 10.1016/j.jpeds.2007.01.033 17452222

[42] Stabouli S Kotsis V Toumanidis S Papamichael C Constantopoulos A Zakopoulos N. White-coat and masked hypertension in children: association with target-organ damage. Pediatr Nephrol (2005) 20(8):1151–5. 10.1007/s00467-005-1979-5 15947982

[43] Sorof JM Poffenbarger T Franco K Portman R. Evaluation of white coat hypertension in children: importance of the definitions of normal ambulatory blood pressure and the severity of casual hypertension. Am J Hypertens (2001) 14(9 Pt 1):855–60. 10.1016/S0895-7061(01)02180-X 11587149

[44] Pall D Juhasz M Lengyel S Molnar C Paragh G Fulesdi B Assessment of target-organ damage in adolescent white-coat and sustained hypertensives. J Hypertens (2010) 28(10):2139–44. 10.1097/HJH.0b013e32833cd2da 20616755

[45] Lande MB Meagher CC Fisher SG Belani P Wang H Rashid M. Left ventricular mass index in children with white coat hypertension. J Pediatr (2008) 153(1):50–4. 10.1016/j.jpeds.2008.01.025 18571535 PMC2516747

[46] Lurbe E Torro I Alvarez V Nawrot T Paya R Redon J Prevalence, persistence, and clinical significance of masked hypertension in youth. Hypertension (2005) 45(4):493–8. 10.1161/01.HYP.0000160320.39303.ab 15767467

[47] Samuels J Ng D Flynn JT Mitsnefes M Poffenbarger T Warady BA Ambulatory blood pressure patterns in children with chronic kidney disease. Hypertension (2012) 60(1):43–50. 10.1161/HYPERTENSIONAHA.111.189266 22585950 PMC3439139

[48] Di Salvo G Castaldi B Baldini L Gala S del Gaizo F D’Andrea A Masked hypertension in young patients after successful aortic coarctation repair: impact on left ventricular geometry and function. J Hum Hypertens (2011) 25(12):739–45. 10.1038/jhh.2010.118 21228825

[49] Mitsnefes M Flynn J Cohn S Samuels J Blydt-Hansen T Saland J Masked hypertension associates with left ventricular hypertrophy in children with CKD. J Am Soc Nephrol (2010) 21(1):137–44. 10.1681/ASN.2009060609 19917781 PMC2799282

[50] Ruiz-Hurtado G Gorostidi M Waeber B Ruilope LM. Ambulatory and home blood pressure monitoring in people with chronic kidney disease. Time to abandon clinic blood pressure measurements? Curr Opin Nephrol Hypertens (2015) 24(6):488–91. 10.1097/MNH.0000000000000162 26371523

[51] Gupta D Chaturvedi S Chandy S Agarwal I. Role of 24-h ambulatory blood pressure monitoring in children with chronic kidney disease. Indian J Nephrol (2015) 25(6):355–61. 10.4103/0971-4065.148305 26664211 PMC4663773

[52] Wuhl E Trivelli A Picca S Litwin M Peco-Antic A Schaefer F Strict blood-pressure control and progression of renal failure in children. N Engl J Med (2009) 361(17):1639–50. 10.1056/NEJMoa0902066 19846849

[53] Seeman T Simkova E Kreisinger J Vondrak K Dusek J Gilik J Improved control of hypertension in children after renal transplantation: results of a two-yr interventional trial. Pediatr Transplant (2007) 11(5):491–7. 10.1111/j.1399-3046.2006.00661.x 17631016

[54] Balzano R Lindblad YT Vavilis G Jogestrand T Berg UB Krmar RT. Use of annual ABPM, and repeated carotid scan and echocardiography to monitor cardiovascular health over nine yr in pediatric and young adult renal transplant recipients. Pediatr Transplant (2011) 15(6):635–41. 10.1111/j.1399-3046.2011.01547.x 21884348

[55] Seeman T Dostalek L Gilik J. Control of hypertension in treated children and its association with target organ damage. Am J Hypertens (2012) 25(3):389–95. 10.1038/ajh.2011.218 22089110

[56] Halbach SM Hamman R Yonekawa K Hanevold C Utility of ambulatory blood pressure monitoring in the evaluation of elevated clinic blood pressures in children. J Am Soc Hypertens (2016) 10(5):406–12. 10.1016/j.jash.2016.02.013 27026571

[57] Flynn JT. Impact of ambulatory blood pressure monitoring on the management of hypertension in children. Blood Press Monit (2000) 5(4):211–6. 10.1097/00126097-200008000-00003 11035862

[58] Gimpel C Wuhl E Arbeiter K Drozdz D Trivelli A Charbit M Superior consistency of ambulatory blood pressure monitoring in children: implications for clinical trials. J Hypertens (2009) 27(8):1568–74. 10.1097/HJH.0b013e32832cb2a8 19550356

[59] Tullus K Secondary Forms of Hypertension. Flynn JIJT Portman RJ, editors. New York: Humana Press (2011).

[60] Brown ML Burkhart HM Connolly HM Dearani JA Cetta F Li Z Coarctation of the aorta: lifelong surveillance is mandatory following surgical repair. J Am Coll Cardiol (2013) 62(11):1020–5. 10.1016/j.jacc.2013.06.016 23850909

[61] Tong F Li ZQ Li L Chong M Zhu YB Su JW The follow-up surgical results of coarctation of the aorta procedures in a cohort of Chinese children from a single institution. Heart Lung Circ (2014) 23(4):339–46. 10.1016/j.hlc.2013.10.060 24239137

[62] Padang R Dennis M Semsarian C Bannon PG Tanous DJ Celermajer DS Detection of serious complications by MR imaging in asymptomatic young adults with repaired coarctation of the aorta. Heart Lung Circ (2014) 23(4):332–8. 10.1016/j.hlc.2013.10.055 24210077

[63] Lee MG Allen SL Kawasaki R Kotevski A Koleff J Kowalski R High prevalence of hypertension and end-organ damage late after coarctation repair in normal arches. Ann Thorac Surg (2015) 100(2):647–53. 10.1016/j.athoracsur.2015.03.099 26138761

[64] McLin VA Anand R Daniels SR Yin W Alonso EM SPLIT Research Group. Blood pressure elevation in long-term survivors of pediatric liver transplantation. Am J Transplant (2012) 12(1):183–90. 10.1111/j.1600-6143.2011.03772.x 21992721

[65] Abu-Elmagd KM Kosmach-Park B Costa G Zenati M Martin L Koritsky DA Long-term survival, nutritional autonomy, and quality of life after intestinal and multivisceral transplantation. Ann Surg (2012) 256(3):494–508. 10.1097/SLA.0b013e318265f310 22868368

[66] Lindenfeld J Page RL II Zolty R Shakar SF Levi M Lowes B Drug therapy in the heart transplant recipient: part III: common medical problems. Circulation (2005) 111(1):113–7. 10.1161/01.CIR.0000151609.60618.3C 15630040

[67] Tainio J Qvist E Miettinen J Holtta T Pakarinen M Jahnukainen T Blood pressure profiles 5 to 10 years after transplant in pediatric solid organ recipients. J Clin Hypertens (Greenwich) (2015) 17(2):154–61. 10.1111/jch.12465 25557075 PMC8031723

[68] Stehlik J Edwards LB Kucheryavaya AY Aurora P Christie JD Kirk R The registry of the International Society for Heart and Lung Transplantation: twenty-seventh official adult heart transplant report – 2010. J Heart Lung Transplant (2010) 29(10):1089–103. 10.1016/j.healun.2010.08.007 20870164

[69] Pruthi J Medkiff KA Esrason KT Donovan JA Yoshida EM Erb SR Analysis of causes of death in liver transplant recipients who survived more than 3 years. Liver Transpl (2001) 7(9):811–5. 10.1053/jlts.2001.27084 11552217

[70] Del Compare ME D’Agostino D Ferraris JR Boldrini G Waisman G Krmar RT. Twenty-four-hour ambulatory blood pressure profiles in liver transplant recipients. Pediatr Transplant (2004) 8(5):496–501. 10.1111/j.1399-3046.2004.00192.x 15367287

[71] O’Sullivan JJ Derrick G Gray J. Blood pressure after cardiac transplantation in childhood. J Heart Lung Transplant (2005) 24(7):891–5. 10.1016/j.healun.2004.05.025 15982619

[72] Baker KS Chow E Steinberger J. Metabolic syndrome and cardiovascular risk in survivors after hematopoietic cell transplantation. Bone Marrow Transplant (2012) 47(5):619–25. 10.1038/bmt.2011.118 21643022 PMC13441451

[73] Hingorani S Renal complications of hematopoietic-cell transplantation. N Engl J Med (2016) 374(23):2256–67. 10.1056/NEJMra1404711 27276563

[74] Bhatia S Francisco L Carter A Sun CL Baker KS Gurney JG Late mortality after allogeneic hematopoietic cell transplantation and functional status of long-term survivors: report from the Bone Marrow Transplant Survivor study. Blood (2007) 110(10):3784–92. 10.1182/blood-2007-03-082933 17671231 PMC2077324

[75] Sulakova T Janda J Cerna J Janstova V Sulakova A Slany J Arterial HTN in children with T1DM – frequent and not easy to diagnose. Pediatr Diabetes (2009) 10(7):441–8. 10.1111/j.1399-5448.2009.00514.x 19500279

[76] Lurbe E Redon J Kesani A Pascual JM Tacons J Alvarez V Increase in nocturnal blood pressure and progression to microalbuminuria in type 1 diabetes. N Engl J Med (2002) 347(11):797–805. 10.1056/NEJMoa013410 12226150

[77] Kang KT Chiu SN Weng WC Lee PL Hsu WC. Analysis of 24-hour ambulatory blood pressure monitoring in children with obstructive sleep apnea: a hospital-based study. Medicine (Baltimore) (2015) 94(40):e1568. 10.1097/MD.0000000000001568 26448004 PMC4616740

[78] Amin R Somers VK McConnell K Willging P Myer C Sherman M Activity-adjusted 24-hour ambulatory blood pressure and cardiac remodeling in children with sleep disordered breathing. Hypertension (2008) 51(1):84–91. 10.1161/HYPERTENSIONAHA.107.099762 18071053

[79] Ng DK Wong JC Chan CH Leung LC Leung SY. Ambulatory blood pressure before and after adenotonsillectomy in children with obstructive sleep apnea. Sleep Med (2010) 11(7):721–5. 10.1016/j.sleep.2009.10.007 20605109

[80] Drager LF Pedrosa RP Diniz PM Diegues-Silva L Marcondes B Couto RB The effects of continuous positive airway pressure on prehypertension and masked hypertension in men with severe obstructive sleep apnea. Hypertension (2011) 57(3):549–55. 10.1161/HYPERTENSIONAHA.110.165969 21242462

[81] Luque-Ramirez M Marti D Fernandez-Duran E Alpanes M Alvarez-Blasco F Escobar-Morreale HF. Office blood pressure, ambulatory blood pressure monitoring, and echocardiographic abnormalities in women with polycystic ovary syndrome: role of obesity and androgen excess. Hypertension (2014) 63(3):624–9. 10.1161/HYPERTENSIONAHA.113.02468 24324038

[82] Takeuchi D Furutani M Harada Y Furutani Y Inai K Nakanishi T High prevalence of cardiovascular risk factors in children and adolescents with Williams-Beuren syndrome. BMC Pediatr (2015) 15:126. 10.1186/s12887-015-0445-1 26384008 PMC4574554

[83] Lama G Graziano L Calabrese E Grassia C Rambaldi PF Cioce F Blood pressure and cardiovascular involvement in children with neurofibromatosis type1. Pediatr Nephrol (2004) 19(4):413–8. 10.1007/s00467-003-1397-5 14991390

[84] Fudge EB Constantacos C Fudge JC Davenport M. Improving detection of hypertension in girls with Turner syndrome using ambulatory blood pressure monitoring. Horm Res Paediatr (2014) 81(1):25–31. 10.1159/000355510 24281046

[85] Akyurek N Atabek ME Eklioglu BS Alp H. Ambulatory blood pressure and subclinical cardiovascular disease in children with Turner syndrome. Pediatr Cardiol (2014) 35(1):57–62. 10.1007/s00246-013-0740-2 23794013

[86] Veltman-Verhulst SM van Rijn BB Westerveld HE Franx A Bruinse HW Fauser BC Polycystic ovary syndrome and early-onset preeclampsia: reproductive manifestations of increased cardiovascular risk. Menopause (2010) 17(5):990–6. 10.1097/gme.0b013e3181ddf705 20551845

[87] Gravholt CH Juul S Naeraa RW Hansen J. Morbidity in Turner syndrome. J Clin Epidemiol (1998) 51(2):147–58. 10.1016/S0895-4356(97)00237-0 9474075

[88] Pober BR Morris CA. Diagnosis and management of medical problems in adults with Williams-Beuren syndrome. Am J Med Genet C Semin Med Genet (2007) 145C(3):280–90. 10.1002/ajmg.c.30139 17639596

[89] Shatat IF Jakson SM Blue AE Johnson MA Orak JK Kalpatthi R. Masked hypertension is prevalent in children with sickle cell disease: a Midwest Pediatric Nephrology Consortium study. Pediatr Nephrol (2013) 28(1):115–20. 10.1007/s00467-012-2275-9 22886281

[90] Lurbe E Agabiti-Rosei E Cruickshank JK Dominiczak A Erdine S Hirth A 2016 European Society of Hypertension guidelines for the management of high blood pressure in children and adolescents. J Hypertens (2016) 34(10):1887–920. 10.1097/HJH.0000000000001039 27467768

[91] Swartz SJ Srivaths PR Croix B Feig DI. Cost-effectiveness of ambulatory blood pressure monitoring in the initial evaluation of hypertension in children. Pediatrics (2008) 122(6):1177–81. 10.1542/peds.2007-3432 19047231

[92] Davis ML Ferguson MA Zachariah JP. Clinical predictors and impact of ambulatory blood pressure monitoring in pediatric hypertension referrals. J Am Soc Hypertens (2014) 8(9):660–7. 10.1016/j.jash.2014.05.011 25065681 PMC4167561

[93] Flynn JT. Ambulatory blood pressure monitoring in children: imperfect yet essential. Pediatr Nephrol (2011) 26(12):2089–94. 10.1007/s00467-011-1984-9 21866381

[94] Kapur G What do I need to know to start ambulatory blood pressure monitoring for children with hypertension? The Section on Nephrology Newsletter. (2013). p. 3–5. Available from: https://www.aap.org/en-us/about-the-aap/Committees-Councils-Sections/Section-on-Nephrology/Documents/NephrologyNewsletter-Spring2013.pdf

